# Supplementary material for: Knowledge, attitude and practice of home management of diarrhea among under-five children in East Africa: A systematic review and meta-analysis
Source: PLoS One. 2024 Feb 23;19(2):e0298801. doi: 10.1371/journal.pone.0298801 (PMC10890776; doi:10.1371/journal.pone.0298801)
Supplement: S6 Fig — (DOCX) [file pone.0298801.s007.docx]

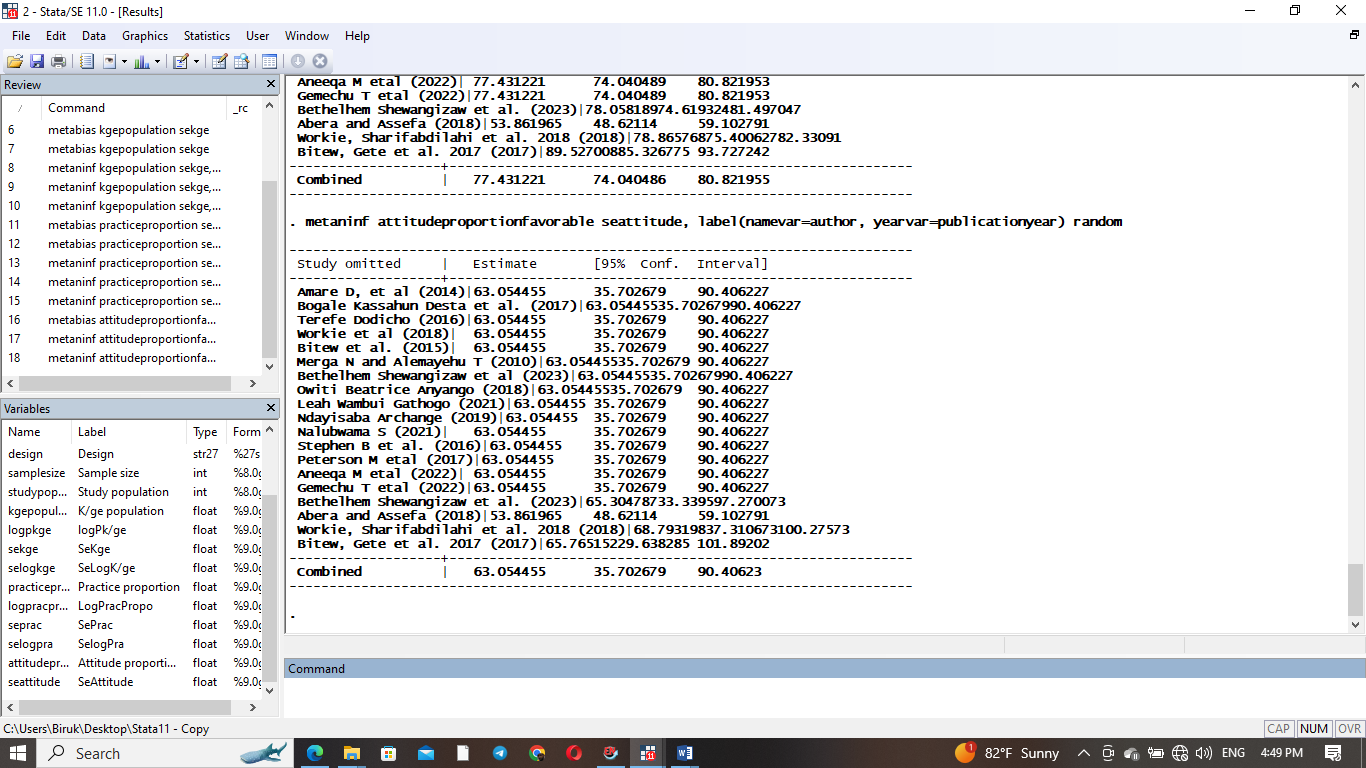


Supplementary Figure 6: Shows sensitivity analysis on attitude towards home based management of diarrhea in by country East Africa
