# Supplementary material for: Knowledge, attitude and practice of home management of diarrhea among under-five children in East Africa: A systematic review and meta-analysis
Source: PLoS One. 2024 Feb 23;19(2):e0298801. doi: 10.1371/journal.pone.0298801 (PMC10890776; doi:10.1371/journal.pone.0298801)
Supplement: Attachment — Submitted filename: PONE-D-23-27980_reviewer comments.pdf [file pone.0298801.s010.pdf]

PLOS ONE

Knowledge, attitude and practice of home management of diarrhea among children in East Africa

--Manuscript Draft--

**Manuscript Number:**	PONE-D-23-27980
**Article Type:**	Research Article
**Full Title:**	Knowledge, attitude and practice of home management of diarrhea among children in East Africa
**Short Title:**	Knowledge, attitude and practice of home management of diarrhea among children in East Africa
**Corresponding Author:**	Biruk Beletew Abate<br>Woldia University<br>Woldia, ETHIOPIA
**Keywords:**	Knowledge; Attitude; Practice; Home-based; Diarrhea; Systematic Review
**Abstract:**	Introduction<br><br>Diarrheal disproportionately affects locations with poor access to health care and safe water and sanitation, and in low-income or marginalized populations. Dehydration due to loss of fluids and electrolytes is estimated to be the main cause of diarrhea-related deaths. Factors of particular importance include care givers knowledge, attitude and practice of management of diarrhea. This systematic review and meta-analysis aimed to assess the pooled estimate of knowledge, attitude and practice of home based management of diarrhea in East Africa.<br><br>Methods<br><br>Using Preferred Reporting Items for Systematic Review and Meta-Analysis (PRISMA) guidelines to search articles from electronic databases (Cochrane library, Ovid platform (Medline, Embase, and Emcare), Google Scholar, CINAHL, PubMed, and institutional repositories in East Africa countries. The last search date was on 01/06/ 2023 G. C. The authors extracted the year of publication, country, study design, knowledge level, attitude level and practice level of home based management of diarrhea. A weighted inverse variance random-effects model was used to estimate the pooled estimates of knowledge, attitude and practice of home based management of diarrhea. The subgroup analysis was done by country, and sample size. Publication bias and sensitivity analysis were also done.<br><br>Results<br><br>A total of 15 articles with (n = 498406 patients) were included for the final analysis. From the random-effects model analysis The pooled prevalence of good practice, good knowledge and favorable attitude towards home based management of diarrhea in East Africa was found to be 52.62 (95% CI: 45.32, 59.92) (95% CI: I2 = 78.3%; p < 0.001), 37.44 (95% CI: 26.99, 47.89) (95% CI: I2 = 89.2%; p < 0.001) and .05 (95% CI: 35.7, 90.41) (95% CI: I2 = 97.8%; p < 0.001) respectively.<br><br>Conclusion and Recommendations<br><br>The level of good knowledge, attitude and practice of home based management of diarrhea in East Africa is found to low. A collaborative effort from different stakeholders to enhance the knowledge, attitude and practice is needed to tackle the burden of diarrhea and its consequences.
**Order of Authors:**	Biruk Beletew Abate
	Molla Azmeraw Bizuayehu
	Alemu Birara Zemariam

Powered by Editorial Manager® and ProduXion Manager® from Aries Systems Corporation

	Addis Wondimagegn
	Biruk Kibret Beletew
	Biruk Gebreegziabher Beletew
**Additional Information:**	
**Question**	**Response**
**Financial Disclosure**<br><br>Enter a financial disclosure statement that describes the sources of funding for the work included in this submission. Review the submission guidelines for detailed requirements. View published research articles from *PLOS ONE* for specific examples.<br><br>This statement is required for submission and **will appear in the published article** if the submission is accepted. Please make sure it is accurate.<br><br>**Unfunded studies**<br>Enter: *The author(s) received no specific funding for this work.*<br><br>**Funded studies**<br>Enter a statement with the following details:<br>• Initials of the authors who received each award<br>• Grant numbers awarded to each author<br>• The full name of each funder<br>• URL of each funder website<br>• Did the sponsors or funders play any role in the study design, data collection and analysis, decision to publish, or preparation of the manuscript?<br>• **NO** - Include this sentence at the end of your statement: *The funders had no role in study design, data collection and analysis, decision to publish, or preparation of the manuscript.*<br>• **YES** - Specify the role(s) played.<br><br>* typeset	The author(s) received no specific funding for this work.
**Competing Interests**<br><br>Use the instructions below to enter a competing interest statement for this submission. On behalf of all authors, disclose any competing interests that could be perceived to bias this	The authors have declared that no competing interests exist.

work—acknowledging all financial support and any other relevant financial or non-financial competing interests.<br><br>This statement is required for submission and **will appear in the published article** if the submission is accepted. Please make sure it is accurate and that any funding sources listed in your Funding Information later in the submission form are also declared in your Financial Disclosure statement.<br><br>View published research articles from *PLOS ONE* for specific examples.<br><br>**NO authors have competing interests**<br><br>Enter: *The authors have declared that no competing interests exist.*<br><br>**Authors with competing interests**<br><br>Enter competing interest details beginning with this statement:<br><br>*I have read the journal's policy and the authors of this manuscript have the following competing interests: [insert competing interests here]*<br><br>* typeset	
**Ethics Statement**<br><br>Enter an ethics statement for this submission. This statement is required if the study involved:<br><br>• Human participants<br>• Human specimens or tissue<br>• Vertebrate animals or cephalopods<br>• Vertebrate embryos or tissues<br>• Field research<br><br>Write "N/A" if the submission does not require an ethics statement.	N/A

Powered by Editorial Manager® and ProduXion Manager® from Aries Systems Corporation

General guidance is provided below. Consult the submission guidelines for detailed instructions. **Make sure that all information entered here is included in the Methods section of the manuscript.**<br><br>**Format for specific study types**<br><br>**Human Subject Research (involving human participants and/or tissue)**<br>• Give the name of the institutional review board or ethics committee that approved the study<br>• Include the approval number and/or a statement indicating approval of this research<br>• Indicate the form of consent obtained (written/oral) or the reason that consent was not obtained (e.g. the data were analyzed anonymously)<br><br>**Animal Research (involving vertebrate animals, embryos or tissues)**<br>• Provide the name of the Institutional Animal Care and Use Committee (IACUC) or other relevant ethics board that reviewed the study protocol, and indicate whether they approved this research or granted a formal waiver of ethical approval<br>• Include an approval number if one was obtained<br>• If the study involved *non-human primates*, add *additional details* about animal welfare and steps taken to ameliorate suffering<br>• If anesthesia, euthanasia, or any kind of animal sacrifice is part of the study, include briefly which substances and/or methods were applied<br><br>**Field Research**<br><br>Include the following details if this study involves the collection of plant, animal, or other materials from a natural setting:<br>• Field permit number<br>• Name of the institution or relevant body that granted permission	
**Data Availability**<br><br>Authors are required to make all data underlying the findings described fully	Yes - all data are fully available without restriction

Powered by Editorial Manager® and ProduXion Manager® from Aries Systems Corporation

available, without restriction, and from the time of publication. PLOS allows rare exceptions to address legal and ethical concerns. See the PLOS Data Policy and FAQ for detailed information.<br><br>A Data Availability Statement describing where the data can be found is required at submission. Your answers to this question constitute the Data Availability Statement and **will be published in the article**, if accepted.<br><br>**Important:** Stating 'data available on request from the author' is not sufficient. If your data are only available upon request, select 'No' for the first question and explain your exceptional situation in the text box.<br><br>Do the authors confirm that all data underlying the findings described in their manuscript are fully available without restriction?	
**Describe where the data may be found in full sentences. If you are copying our sample text, replace any instances of XXX with the appropriate details.**<br><br>• If the data are **held or will be held in a public repository**, include URLs, accession numbers or DOIs. If this information will only be available after acceptance, indicate this by ticking the box below. For example: *All XXX files are available from the XXX database (accession number(s) XXX, XXX.*).<br>• If the data are all contained **within the manuscript and/or Supporting Information files**, enter the following: *All relevant data are within the manuscript and its Supporting Information files.*<br>• If neither of these applies but you are able to provide **details of access elsewhere**, with or without limitations, please do so. For example:<br><br>*Data cannot be shared publicly because of [XXX]. Data are available from the XXX Institutional Data Access / Ethics*	The data underlying the results presented in the study are available from corresponding author

Powered by Editorial Manager® and ProduXion Manager® from Aries Systems Corporation

Committee (contact via XXX) for researchers who meet the criteria for access to confidential data.<br><br>*The data underlying the results presented in the study are available from (include the name of the third party and contact information or URL).*<br>• This text is appropriate if the data are owned by a third party and authors do not have permission to share the data.<br><br>* typeset	
Additional data availability information:	

Powered by Editorial Manager® and ProduXion Manager® from Aries Systems Corporation

Click here to access/download;Manuscript;KAP for home managment of diarrhea SRM.docx

Knowledge, attitude and practice of home management of diarrhea among children in East Africa

Biruk Beletew Abate[1*], Molla Azmeraw Bizuayehu[3], Alemu Birara Zemariam[3], Addis Wondimagegn[5], Gebremeskel Kibret Abebe[5], Freweyni Gebreegziabher Araya[3]

Authors Affiliation

[1*]Assistant Professor in Pediatrics and Child Health Nursing, College of Health Science, Woldia University, Ethiopia

[2]MSc in Integrated Clinical and Community Mental Health, College of Health Science, Woldia University, Ethiopia

[3]MSc in Pediatrics and Child Health Nursing, College of Health Science, Woldia University, Ethiopia

[5]MSc in Emergency Medicine and Critical Care Nursing, College of Health Science, Woldia University, Ethiopia

***Corresponding Author: Biruk Beletew Abate**: Assistant Professor, Woldia University Ethiopia; Email: birukkelemb@gmail.com: Cell Phone: +251922898070

Abstract

Introduction: Diarrheal disproportionately affects locations with poor access to health care and safe water and sanitation, and in low-income or marginalized populations. Dehydration due to loss of fluids and electrolytes is estimated to be the main cause of diarrhea-related deaths. Factors of particular importance include care givers knowledge, attitude and practice of management of diarrhea. This systematic review and meta-analysis aimed to assess the pooled estimate of knowledge, attitude and practice of home based management of diarrhea in East Africa.

Methods: Using Preferred Reporting Items for Systematic Review and Meta-Analysis (PRISMA) guidelines to search articles from electronic databases (Cochrane library, Ovid platform (Medline, Embase, and Emcare), Google Scholar, CINAHL, PubMed, and

institutional repositories in East Africa countries. The last search date was on 01/06/ 2023 G. C. The authors extracted the year of publication, country, study design, knowledge level, attitude level and practice level of home based management of diarrhea. A weighted inverse variance random-effects model was used to estimate the pooled estimates of knowledge, attitude and practice of home based management of diarrhea. The subgroup analysis was done by country, and sample size. Publication bias and sensitivity analysis were also done.

Results: A total of 15 articles with (n = 498406 patients) were included for the final analysis. From the random-effects model analysis The pooled prevalence of good practice, good knowledge and favorable attitude towards home based management of diarrhea in East Africa was found to be 52.62 (95% CI: 45.32, 59.92) (95% CI: I2 = 78.3%; $p < 0.001$), 37.44 (95% CI: 26.99, 47.89) (95% CI: I^2 = 89.2%; $p < 0.001$) and .05 (95% CI: 35.7, 90.41) (95% CI: I^2 = 97.8%; $p < 0.001$) respectively.

Conclusion and Recommendations: The level of good knowledge, attitude and practice of home based management of diarrhea in East Africa is found to low. A collaborative effort from different stakeholders to enhance the knowledge, attitude and practice is needed to tackle the burden of diarrhea and its consequences.

Key words: Knowledge, Attitude, Practice, Home-based, Diarrhea, Systematic Review

Introduction

Diarrhea is passing of three or more loose or liquid stools per day, or more frequently than is normal for the individual [1]. Diarrhea can be acute watery, bloody/dysentery or persistent. As diarrhea causes loss of body fluid and electrolytes it leads to dehydration which is the common cause of death, malnutrition, and other severe complications [2]. It is one of the leading causes of morbidity and mortality in children under the age of 5 all over the world [3, 4]. Diarrheal diseases disproportionately affect locations with poor access to health care and safe water and sanitation, and in low-income or marginalized populations [5]. Dehydration due to loss of fluids and electrolytes is estimated to be the main cause of diarrhea-related deaths causing 60%–70% of diarrhea-related deaths in children under the age of

5[6-8]. Factors of particular importance include care givers knowledge about causes of diarrhea and the associated dangers signs, prevention of dehydration during diarrheal episodes through the use of Oral Rehydration Salts[9, 10]. Therefore, it is strongly recommended to provide prompt rehydration therapy to patients with diarrhea.

Globally in 2019, over 5 million under five children died and 484,000 were due to diarrheal disease which could be easily prevented and treated. Sub-Saharan Africa countries take more than half of the global burden of under-five mortality. ~~As of the year~~ 2000 to 2019, morbidity and mortality rate due to diarrhea ~~has~~ decreased from 12.6% to 9.1% by means of improvement in safe water, sanitation, vaccine, and treatment coverage. Despite this, diarrhea is still the leading cause of under-five mortality worldwide, especially in Africa [11-13] . Unsafe water supply, sanitation, and nutrition, are the main contributing factors for the highest prevalence of diarrhea. Even ~~if~~ worldwide safe water usage and sanitation reached 71% and 39%, Africa ~~is having a drawback on these needs~~ (44% safely managed drinking-water services, and 21% safely managed sanitation services). Globally in 2016, almost 0.9 million deaths were due to unsafe drinking water, unsafe sanitation, and lack of hygiene, in which diarrhea accounted for over 470,000 deaths of under five years children [12, 14]. Diarrhea in children can be managed at home before it becomes ~~sever~~ and problematic. Home based management of diarrhea is a protocol developed to easily manage diarrhea and prevent the complications with a minimum cost. The management includes: increasing fluid and food intake including breast feeding, giving oral rehydration solution, and rest. Inappropriate home management practice can worsen a child's condition and even cause death [15, 16]. Effective therapies for diarrhea exist, but children in poor rural communities often do not have access to formal healthcare[17, 18], and coverage of these interventions remains low in many developing countries[19]. The level of home management practice of diarrhea is poor. Similarly, their practice to use universal popular ORS in preventing dehydration due to diarrhea is also very low[3]. Inadequate treatment of diarrhea, which is often caused by financial issues, geographic inaccessibility to well-equipped health facilities, and shortage of qualified health professionals and essentials drugs, is one of the risk factors of diarrhea-related mortality[20].

Family plays the major role in the treatment and surviving chance of a child with diarrhea. For the effective management of diarrhea at home, caregivers should have a basic knowledge and good attitude toward home based management. In Africa different studies have conducted regarding Knowledge, attitude, and practice on home based management of diarrhea and they lack consistency (knowledge ranges from 36.6% to 67%, attitude 45.1% to 94.4%, and practice 12% to 58%). As per the investigators knowledge there are no systematic review and meta-analysis done to address the inconsistence reports from Africa. There are limited studies in the study area which investigated the level of home-based diarrhea management practice among the caregivers of children under the age of 5. Even though the bundling of these drugs was vital in the treatment of diarrhea for under-five children the prevalence was minimal in various countries as in Bangladesh, 49%; Tanzania,18%; Nigeria, 10% and Sudan, 15%. According to a study conducted in Ethiopia, about seven out of ten caregivers had utilized this bundled drugs[21]. Also, the Ethiopian EDHS-2016 report indicated that only 17% received a combination of ORS and zinc[22].

A number of significant public health benefits from introduction of co-packaged ORS and zinc have already been demonstrated, including: Increased uptake and coverage of ORS and zinc (as a combination therapy and as individual components dispensed together), reducing the risk of severe health consequences of chronic diarrhea and stunting, acute diarrhea, and zinc deficiency among children, improved adherence to the combined therapy of ORS and zinc, improved adherence to/preparation of individual components (e.g., correct concentration of prepared ORS, completion of a full course of zinc), improved dispensing practices by health care workers. Co-packaging also offers cost savings, as promoting use of both ORS and zinc can bring down the overall cost of long-term health care and financial burden to families and communities[23]. Despite the existence of these simple, inexpensive, and lifesaving treatments, access to ORS and zinc remains a challenge in low-resource settings, and the rate of co-administration is extremely low. Co-package of ORS and zinc could be delivered by community health workers, or directly to households through campaigns or Child Health Days. Pregnant women could also receive such kits during antenatal care visits. Use of ORS and Zink together is optimal for diarrhea management, yet awareness, uptake, and demand remains low. Thus, the purpose of this study is to evaluate whether co-packing of zinc with ORS using

a plastic pouch can enhance the joint adherence to diarrhea treatment or not. The study also compared the CE (cost per unit of a defined health outcome of ‘central’ and ‘health center (HC)’ level bundling approaches as a means to provide empirical evidence for decision.

The role of the family, especially the mother, is vital in health promotion, disease prevention, and patient care. In the actions mothers take, the minimum required is a brief and superficial examination of the dehydrated child and the amount and type of liquid fed to him or her in the case of diarrhea, even these actions are vital for pediatric welfare[24]. Proper home-based management can reduce morbidity and mortality due to diarrhea in children under the age of 5. Delivery of care through community health workers (CHWs**)** can increase coverage of specific treatments [25] and lead to substantial reductions in child mortality[26, 27]. The World Health Organization (WHO) and the United Nations Children’s Fund (UNICEF) recommend integrated community case management (iCCM) of diarrhea[28]. However, experience with large scale implementation of iCCM remains limited[29]. Few rigorous assessments of iCCM implementation have been conducted, and the limited evidence on quality of care is mixed [30-33]. This systematic review and meta-analysis aimed to assess the pooled estimate of knowledge, attitude and practice of home based management of diarrhea in East Africa.

Methods

Search strategy

This systematic review and meta-analysis review assessed studies that provide data on the knowledge, attitude and practice of home based management of diarrhea and /or its determinants in East Africa context. We searched these articles from the following databases: Cochrane library, Ovid platform (Medline, Embase, and Emcare), Google Scholar, CINAHL, PubMed, and institutional repositories in East Africa countries on 01/06/ 2023 G. C. The search in all database included keywords that are the combinations of population, condition/outcome, and context. A snowball searching for the references of relevant papers for linked articles was also performed. The following search map was applied: “knowledge”, “awareness”, “attitude”, “perception” “practice”, “home-based”, “community-based”, “management”, “Diarrhea”, “East-Africa” on PubMed database. These search terms were further paired with the names of East-African countries. Using those key terms, we used the Boolean operator "OR" (to connect key terms/phrases within the same concept), "AND"

(to connect key terms /phrases between two concepts), and "NOR" to filter out. In addition, we used truncation (*), adjacency searching (**ADJn)**, and wildcard symbols to find variations in spelling and variant word endings on the Ovid databases. Moreover, we applied relevant limits (filters) such as a limit to human studies only. The sample search strategy for Medline is attached in the appendix section of this manuscript.

Study selection and screening

The retrieved studies were exported to Endnote version 8 reference managers to remove duplicate studies. Two investigators (BB and MA) independently screened the selected studies using article's title and abstracts before retrieval of full-text papers. We used pre-specified inclusion criteria to further screen the full-text articles. Disagreements were discussed during a consensus meeting with other reviewers (AB and AW) for the ~~fin~~ selection of studies to be included in the systematic review and meta-analysis. The retrieved studies were imported in covidence platform to remove duplicate studies and to do the whole screening process.

Inclusion and exclusion criteria

Studies that assess the level of knowledge, attitude and practice of home based management of diarrhea among children in East-Africa context were included. Citations without abstract and/or full text, anonymous reports, editorials, and qualitative studies were excluded from the analysis.

Quality assessment

The authors appraised the quality of the studies by using the Joanna Briggs Institute (JBI) quality appraisal checklist [34]. There was a team of four reviewers and the papers were split amongst the team. Each paper was then assessed by two reviewers and any disagreements were discussed with the third and the fourth reviewers. Studies were considered as low risk or good quality when it scored 4 and above for all designs, whereas the studies scored 3 and below were considered as high risk or poor quality and excluded (Table S2).

Data extraction

The authors developed a data extraction form on the excel sheet and the following data were extracted for eligible studies: year of publication, country, study design, knowledge level, attitude level and practice level of home based management of diarrhea. The data extraction sheet was piloted using 4 papers randomly, and it was adjusted after piloted the template. Two of the authors (BB and GK) extracted the data using the extraction form in collaboration. The third and fourth (AW and FG) authors checked the correctness of the data independently. Any disagreements between reviewers were resolved through discussions with third and fourth reviewers when required. The mistyping of data was resolved through crosschecking with the included papers.

Synthesis of results

The authors transformed the data to STATA 17 for analysis after it was extracted in an excel sheet considering knowledge level, attitude level and practice level of home based management of diarrhea. We pooled the overall prevalence estimates of knowledge, attitude and practice by a random effect meta-analysis model. We examined the heterogeneity of effect size using the Q statistic and the I^2 statistics. In this study, the I^2 statistic value of zero indicates true homogeneity, whereas the value 25%, 50%, and 75% represented low, moderate and high heterogeneity, respectively. Subgroup analysis was done by the study country. Sensitivity analysis was employed to examine the effect of a single study on the overall estimation. Publication bias was checked by the funnel plot and more objectively through Egger's regression test.

Results

A total of 5394 studies were identified; 4380 from different databases and 14 from other sources. After duplication removed, a total of 2,431 articles remained (1963 removed by duplication). Finally, 206 studies were screened for full-text review, and 15 articles with (n = 498406 patients) were included for the final analysis (Fig. 1).

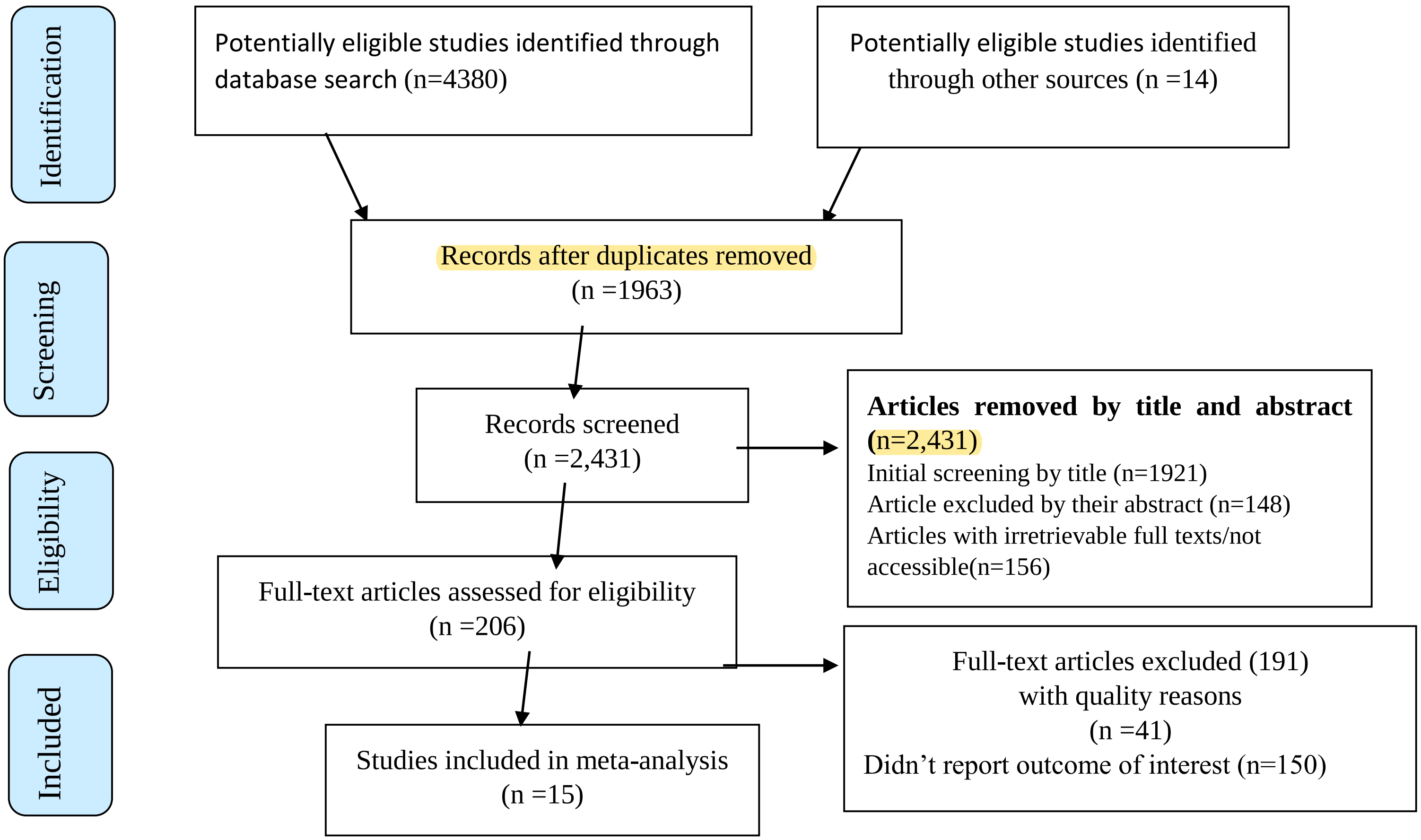

Figure 1: PRISMA –adapted flow diagram showed the results of the search and reasons for exclusion[35].

Characteristics of included studies

Table 1: summarizes the characteristics of the 15 included studies in this systematic review and meta-analysis [36-49]. Two studies were from Ethiopia,[36, 42] two from Uganda,[37, 39] two from Tanzania,[38, 40] three from Egypt,[41, 46, 47] one from Cameroon,[48] one from Nigeria,[49] two from South Africa,[43, 45] and one from Sudan.[44] (Table 1).

Table 1: Distribution of included studies on the prevalence and characteristics of cerebral palsy in Africa, 2023

Sr No	Authors	Year	Country	Study design	Sample size	Prevalence (%)	Characteristics/types of cerebral palsy						
							Spastic	Quadriplegic	Ataxic	Dyskinestic	Bilateral	Unilateral	Mixed
**1.**	Labena, F. et al[36]	2021	Ethiopia	Cross-sectional	402	..	88.9	62.5	1.4	7.6	9.7	27.1	2.1
**2.**	Kakooza-Mwesige, A. et al[37]	2016	Uganda	Cross-sectional	135	..	23		9.6	12.6	45.9	23.7	8.1
**3.**	Ngassa, E.A., et al [38]	2018	Tanzania	Cross-sectional	120	17.77	..	..	..	..	..	..	..
**4.**	Kakooza-Mwesige, A., et al [39]	2017	Uganda	Cross-sectional	31756	2.9	..	..	2	9	40	46	2
**5.**	Karumuna, J. et al[40]	1990	Tanzania	Cohort	100	10	..	..	9	..	20	15	5
**6.**	El-Tallawy, HN., et al [41]	2008	Egypt	Cross-sectional	25540	2.03	65.4	42.5	3.8	3.8	9.6	13.5	26.9
**7.**	El-Tallawy, H.N., et al [46]	2010	Egypt	Cross-sectional	25540	2.03	..	..	..	..	..	..	..
**8.**	Abas, O. et al[47]	2017	Egypt	Cross-sectional	198776	1	72.5	30.3	7	16	48.27	21.4	
**9.**	El-Tallawy, H.N., et al[41]	2014	Egypt	Cross-sectional	12788	3.6	72.5		3.9	..	..	..	23.5
**10.**	Mangamba, D.K., et al [48]	2022	Cameroon	Cross-sectional	4064	4.86	..	..	..	..	..	..	..
**11.**	R Duke, et al.[49]	2020	Nigeria	Cross-sectional	171200	2.3	69.9	..	9.8	4.6	60.2	39.8	8.3
**12.**	Tsige, S., et al.[42]	2021	Ethiopia	Cross-sectional	207	..	..	..	3.4	10.4	60.4	21.8	4
**13.**	Coombe, H.J. et al. [43]	2017	South Africa	Cross-sectional	94	..	..	27.6	9.6	29.8	12.8	16	2.1
**14.**	Salih, K. et al. [44]	2020	Sudan	Cohort	108	..	..	43.5	3.7		13.9	25.9	2.8
**15.**	Couper, J.[45]	2002	South Africa	Cross-sectional	2 036	10	..	..	..	..	..	..	..

Knowledge level on home based management of diarrhea

Regarding knowledge level of participants on home based management of diarrhea, the pooled prevalence of good knowledge from the random-effects model analysis in Africa was found to be 52.62 (95% CI: 45.32, 59.92) (~~95% CI:~~ I^2 = 78.3%; p < 0.001) (Fig. 2). **S**ubgroup analysis was ~~done through~~ stratified by country. Based on this, the pooled prevalence of good knowledge was revealed 55.78(47.77, 63.79) in Ethiopia, 42.74(33.65, 51.83) in Kenya, and 44.4 (14.16, 74.64) in Rwanda (Fig. 3).

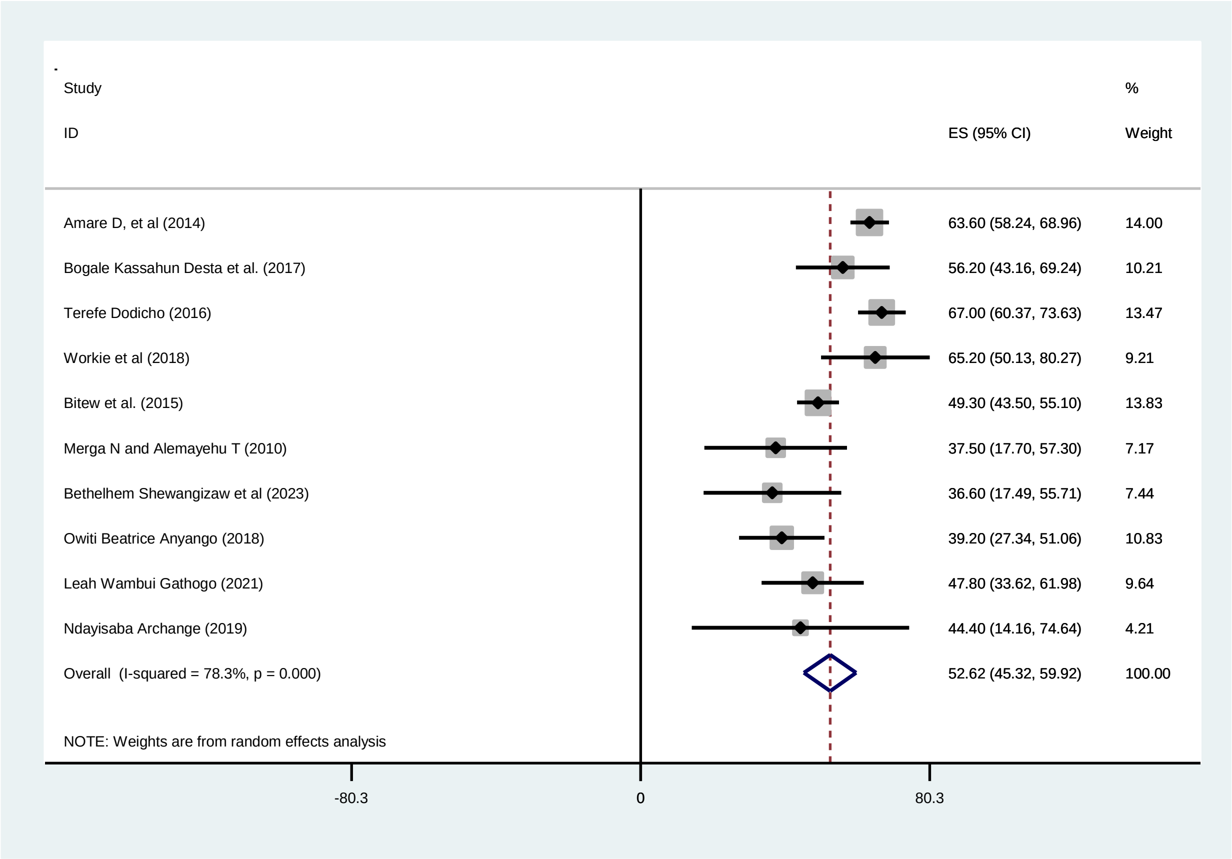

Figure 2 : Forest plot shows knowledge level on home based management of diarrhea in East Africa

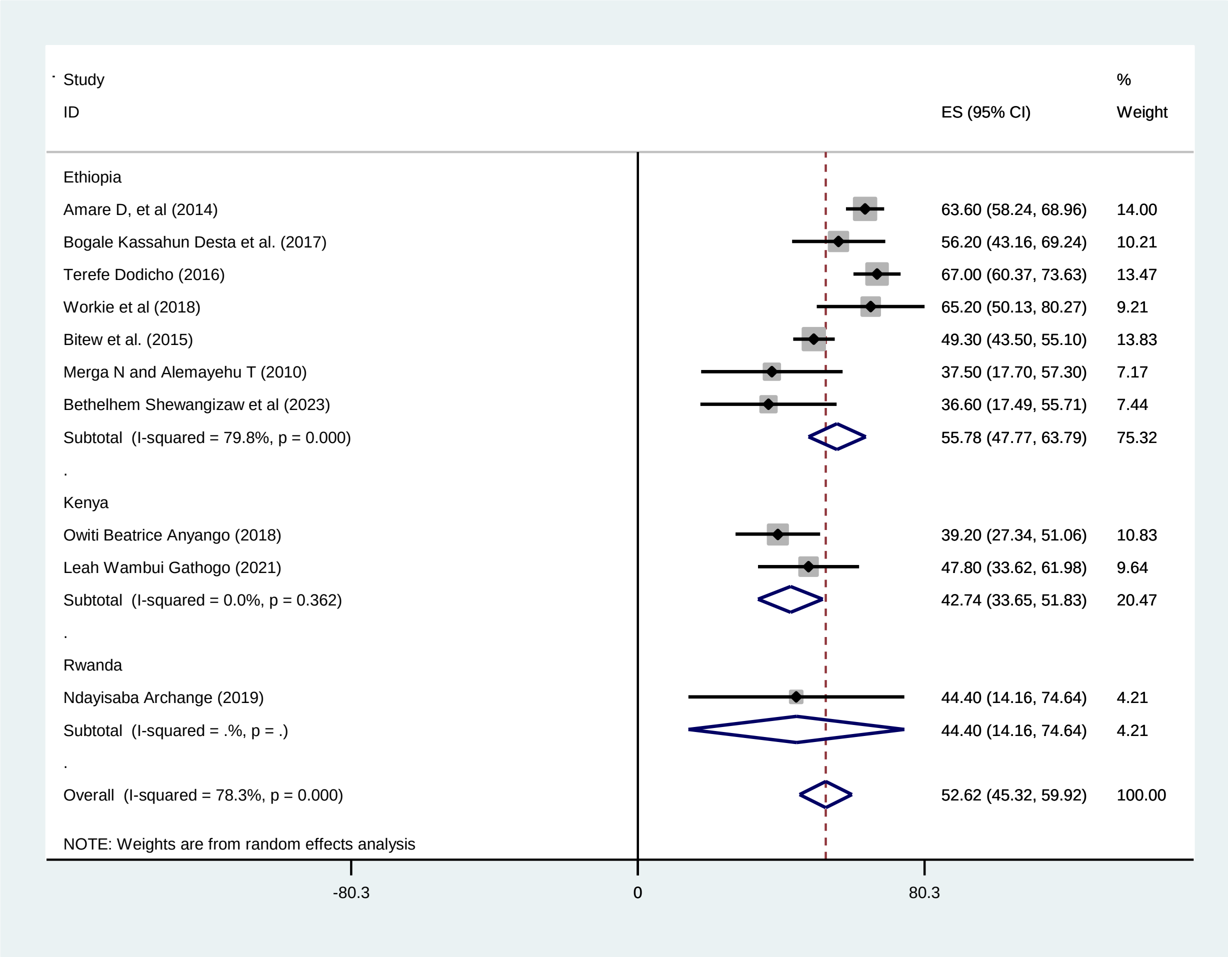

Figure 3: Subgroup analysis knowledge level on home based management of diarrhea in by country East Africa

Publication bias: A funnel plot showed symmetrical distribution. The Egger's regression test-value was 0·149, which indicated ~~that,~~ the absence of publication bias. As a result, we didn't conduct trim and fill analysis (Supplementary Figure 1). **Sensitivity analysis:** We also employed a leave-one-out sensitivity analysis to identify the potential source of heterogeneity in the analysis of the pooled

magnitude of knowledge on home based management of diarrhea. The results of this sensitivity analysis showed that the findings were not dependent on a single study (Supplementary Figure 2).

Practice level on home based management of diarrhea

The pooled prevalence of good practice from the random-effects model analysis in Africa was found to be 37.44 (95% CI: 26.99, 47.89) (~~95% CI:~~ $I^2 = 89.2\%$; $p < 0.001$) (Fig. 4). **S**ubgroup analysis was ~~done through~~ stratified by country. Based on this, the pooled prevalence of good practice was found to be 47.83(42.04,53.61) in Ethiopia, 17.00(9.44,24.56) in Kenya, 47.45(16.95, 78.05) in Rwanda, 25.00(13.37, 36.64) in Uganda, and 12.00(1.10,25.10) in South Sudan (Fig. 5).

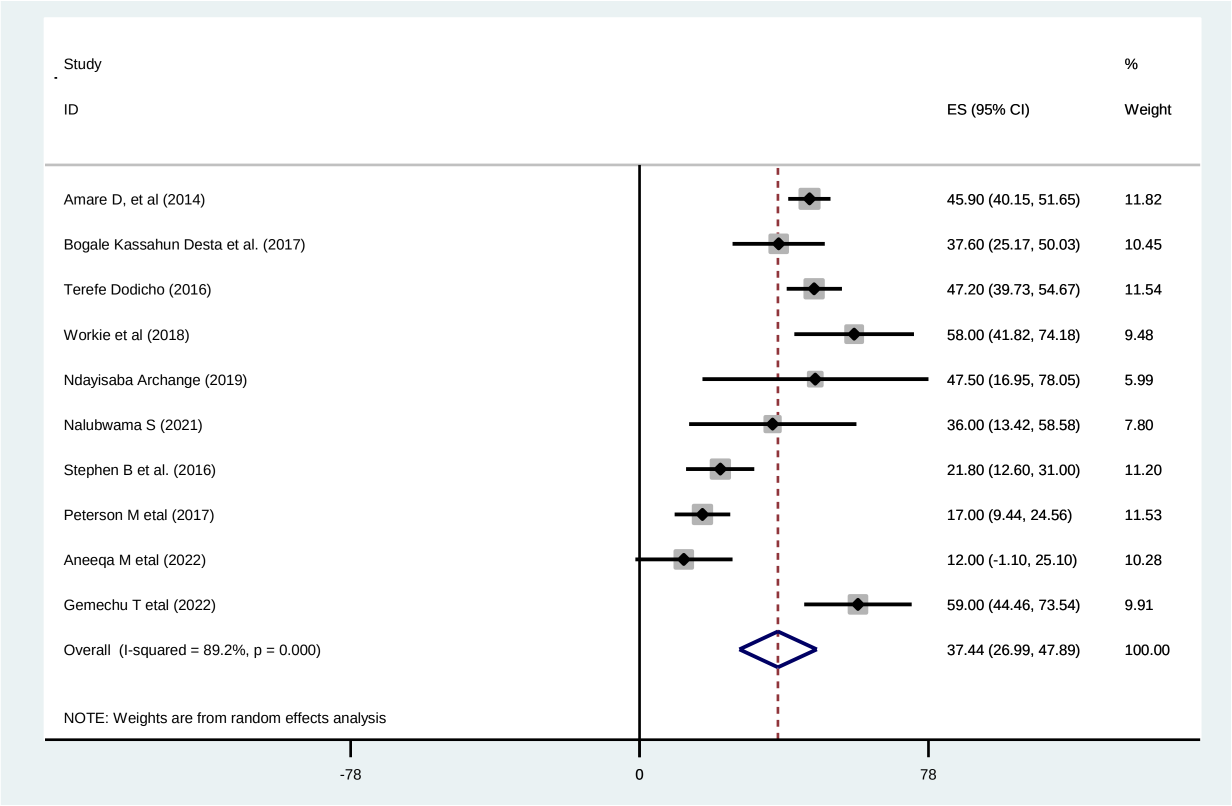

Figure 4 : Forest plot shows practice level of home based management of diarrhea in East Africa

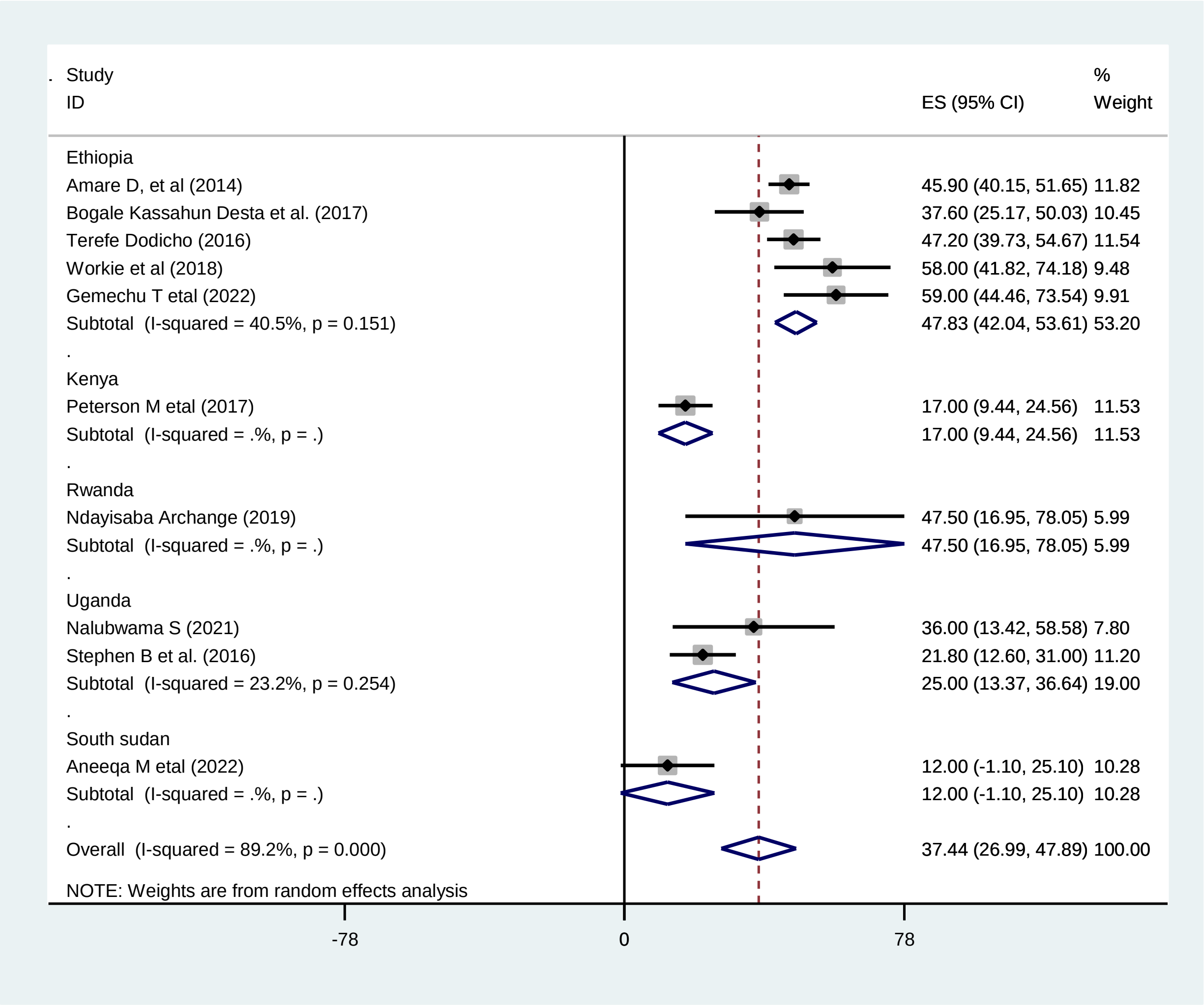

Figure 5: Subgroup analysis shows practice level of home based management of diarrhea by country in East Africa

Publication bias: A funnel plot showed symmetrical distribution. The Egger's regression test-value was 0·965, which indicated ~~that,~~ the absence of publication bias. As a result, we didn't conduct trim and fill analysis (Supplementary Figure 3). **Sensitivity analysis:** We

also employed a leave-one-out sensitivity analysis to identify the potential source of heterogeneity in the analysis of the practice of home based management of diaarrhea. The results of this sensitivity analysis showed that the findings were not dependent on a single study (Supplementary Figure 4).

Level of attitude towards home based management of diarrhea

The pooled prevalence of favorable attitude from the random-effects model analysis in Africa was found to be 63.05 (95% CI: 35.7, 90.41) (~~95% CI:~~ $I^2 = 97.8\%$; $p < 0.001$) (Fig. 6).

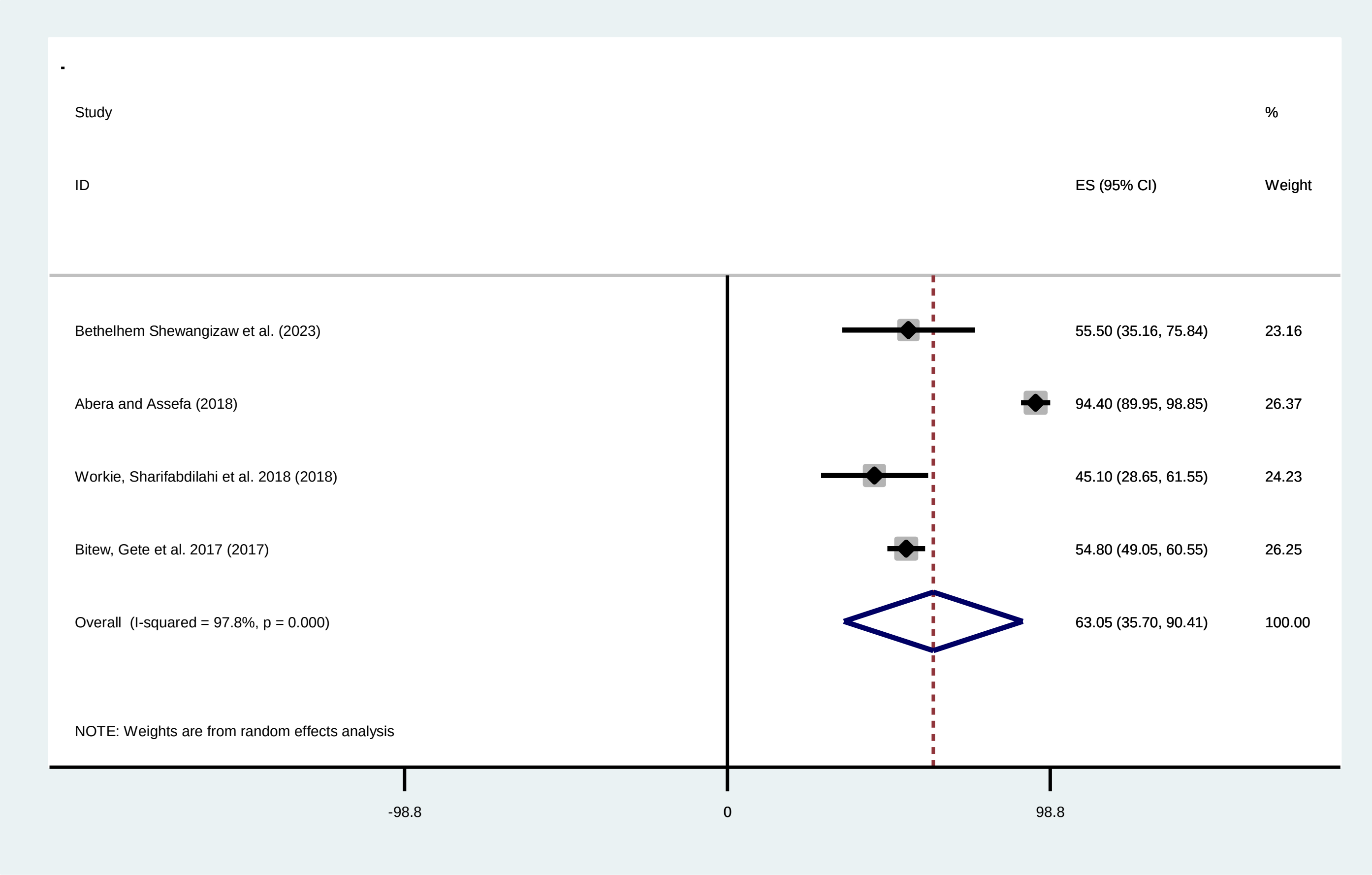

Figure 6 : Forest plot shows attitude level towards home based management of diarrhea in East Africa

Publication bias: A funnel plot showed symmetrical distribution. The Egger's regression test-value was 0·965, which indicated that, the absence of publication bias. As a result, we didn't conduct trim and fill analysis (Supplementary Figure 5). **Sensitivity analysis:** We also employed a leave-one-out sensitivity analysis to identify the potential source of heterogeneity in the analysis of the attitude towards home based management of diarrhea. The results of this sensitivity analysis showed that the findings were not dependent on a single study (Supplementary Figure 6).

Discussion

Over the past several decades, there has been a significant decline in child mortality throughout the world. From 12.6 million deaths of children under five in 1990, the total number fell to 5.6 million in 2016[50]. Diseases that are easily preventable or curable with effective, affordable therapies account for the majority of fatalities among children under the age of five. The great majority of under-five deaths worldwide are caused by infectious illnesses and newborn complications[50]. A concerted effort is needed to put an end to all avoidable infant and child deaths under the age of five in order to achieve the sustainable development goals (SDG) aim of decreasing under-five mortality to 25 per 1000 live births[50].

Diarrhea is one of the major contributors of child morbidity and mortality[51]. Reduced morbidity and mortality load from diarrhea in young children, especially those under the age of five, can be achieved by caregivers having the correct understanding on the appropriate steps to take. The ability of the caregivers to detect the problem and appropriately respond to it is the first step in any appropriate treatment of a sick child. In terms of the onset of complications (especially dehydration) and/or the incidence of mortality, home remedies for the treatment of diarrhea represent a form of "first aid" that are crucial in deciding the course of diseases connected with this symptom[52].

The World Health Organization (WHO) have recognized and supported home-based oral therapy in the management of diarrhea to lessen the impact of diarrhea, particularly on children[51]. Despite this strategy, the pooled knowledge, attitude and practice of care givers of children on home-based management of diarrhea were 52.62%, 60% and 37.4%, respectively in this systematic and meta-analysis study. The finding was lower than the previous study in Afghanistan knowledge (84.8%), attitude (73.6%) and practice (80.7%)[53]. To our search knowledge, this systematic review and meta-analysis is the first of its kind aimed to assess the maternal/ caregivers' knowledge on home-based management of diarrhea in East Africa. Accordingly, the estimated pooled prevalence of good knowledge on home-based diarrhea management was found to be 52.62% (95% CI: 45.32, 59.92). This finding was in line with studies conducted in Nigeria [54], Yemen [55], and India [56], which reported that 59.2%, 51.2%, and 58.7% of mothers had good knowledge

on home-based management of diarrhea, respectively. This might be due to the effect of different projects implemented by WHO that provided a comprehensive community knowledge via health extension worker and different health care providers, which created a common understanding across different nations and populations regarding the home management of diarrhea. However, the current finding was higher than a study conducted in Namibia (36%) [57]. This difference could be attributed to the disparity in socio-economic and socio-demographic characteristics of the included population, such as the educational status of the included mothers'. Besides, this difference might be due to the sample size and outcome measurement variation across the study. On the other hand, the current finding was lower than studies conducted in Nigeria (95.4%) [58] and Pakistan (72.1%) [59]. The possible reason for this difference is due to population variation since the study conducted in Pakistan was among urban slum populations, who are expected to have better exposure to different health information and a good level of understanding regarding home-based management of diarrhea.

This poor knowledge, attitude and practice of home based management of diarrhea in East Africa might be somewhat influenced by their living situation, level of economic development, frequency of health information dissemination and population quality overall[60]. Furthermore, most African population were settled in rural areas where they may not be accessed for information communication and educations services on health care seeking behaviors of the community. On the other side, literacy rates and public awareness via mass media and other formal forms of communication or publicity are relatively low to break the existing culture and belief with in the community, which may account for the discrepancy. The finding implies the requirement of fair and equitable dissemination of health-related information which boosts home based management of diarrhea.

Conclusion and Recommendations

The level of good knowledge, attitude and practice of home based management of diarrhea in East Africa is found to low compared to previous study findings and WHO recommendation. A collaborative effort from different stakeholders to enhance the knowledge, attitude and practice is needed to tackle the burden of diarrhea and its consequences.

Reference

1. Mumtaz, Y., M. Zafar, and Z. Mumtaz, *Knowledge attitude and practices of mothers about diarrhea in children under 5 years.* Journal of the Dow University of Health Sciences (JDUHS), 2014. **8**(1): p. 3-6.
2. Organization, W.H., *Water, sanitation, hygiene and health: a primer for health professionals*. 2019, World Health Organization.
3. Liu, L., et al., *Global, regional, and national causes of child mortality: an updated systematic analysis for 2010 with time trends since 2000.* The lancet, 2012. **379**(9832): p. 2151-2161.
4. Hassan, M., *Knowledge, attitude and practice survey of oral rehydration therapy at the level of health centers in Karari locality*. 2015.
5. Troeger, C., et al., *Estimates of global, regional, and national morbidity, mortality, and aetiologies of diarrhoeal diseases: a systematic analysis for the Global Burden of Disease Study 2015.* The Lancet infectious diseases, 2017. **17**(9): p. 909-948.
6. Ogunrinde, O.G., et al., *Knowledge, attitude and practice of home management of childhood diarrhoea among caregivers of under-5 children with diarrhoeal disease in Northwestern Nigeria.* Journal of tropical pediatrics, 2012. **58**(2): p. 143-146.
7. Prüss-Ustün, A., et al., *Burden of disease from inadequate water, sanitation and hygiene for selected adverse health outcomes: an updated analysis with a focus on low-and middle-income countries.* International journal of hygiene and environmental health, 2019. **222**(5): p. 765-777.
8. Organization, W.H., *The treatment of diarrhoea: a manual for physicians and other senior health workers*. 2005, World Health Organization.
9. Eshete, A., *Assessment of Knowledge, Practice and Utilaization of Oral Rehayderation Therapy for Acute Watery Diarrhoeal Disease Case Management among Mothers (Caregivers') of Under-Five Children in Assela Town, Ethiopia*. 2015, Addis Ababa University.
10. Ng, E., *The pandemic of hate is giving COVID-19 a helping hand.* The American Journal of Tropical Medicine and Hygiene, 2020. **102**(6): p. 1158-1159.
11. Rushton, J., et al., *Initiation of global burden of animal diseases programme.* The Lancet, 2018. **392**(10147): p. 538-540.
12. Liu, L., et al., *Global, regional, and national causes of under-5 mortality in 2000–15: an updated systematic analysis with implications for the Sustainable Development Goals.* The Lancet, 2016. **388**(10063): p. 3027-3035.
13. UNICEF and W.H. Organization, *Levels & trends in child mortality estimates developed by the UN Inter-Agency Group for Child Mortality Estimation.* 2010.
14. Organization, W.H., *World health statistics overview 2019: monitoring health for the SDGs, sustainable development goals*. 2019, World Health Organization.
15. Organization, W.H., *The management and prevention of diarrhoea: practical guidelines*. 1993: World Health Organization.
16. Kiplagat, A., et al., *Factors influencing the implementation of integrated management of childhood illness (IMCI) by healthcare workers at public health centers & dispensaries in Mwanza, Tanzania.* BMC public health, 2014. **14**(1): p. 1-10.
17. Kahabuka, C., *Care seeking and management of common childhood illnesses in Tanzania: Questioning the quality of primary care services*. 2013: The University of Bergen.
18. Victora, C.G., et al., *Applying an equity lens to child health and mortality: more of the same is not enough.* The Lancet, 2003. **362**(9379): p. 233-241.

19. Bhutta, Z.A., et al., *Countdown to 2015 decade report (2000–10): taking stock of maternal, newborn, and child survival.* The lancet, 2010. **375**(9730): p. 2032-2044.
20. Mokomane, M., et al., *The global problem of childhood diarrhoeal diseases: emerging strategies in prevention and management.* Therapeutic advances in infectious disease, 2018. **5**(1): p. 29-43.
21. Kodgule, R. and S. Salvi, *Exposure to biomass smoke as a cause for airway disease in women and children.* Current opinion in allergy and clinical immunology, 2012. **12**(1): p. 82-90.
22. Po, J.Y., J.M. FitzGerald, and C. Carlsten, *Respiratory disease associated with solid biomass fuel exposure in rural women and children: systematic review and meta-analysis.* Thorax, 2011. **66**(3): p. 232-239.
23. R., R., *Emulating Commercial, Private-Sector Value-Chains to Improve Access to ORS and Zinc in Rural Zambia: Evaluation of the Colalife Trial. Dissertation submitted to The Johns Hopkins University in conformity with the requirements for the degree of Doctor of Public Health. 2016. https://jscholarship.library.jhu.edu/handle/1774.2/39229.* 2016.
24. Ene-Obong, H.N., C.U. Iroegbu, and A.C. Uwaegbute, *Perceived causes and management of diarrhoea in young children by market women in Enugu State, Nigeria.* Journal of Health, Population and Nutrition, 2000: p. 97-102.
25. Dawson, P., et al., *From research to national expansion: 20 years' experience of community-based management of childhood pneumonia in Nepal.* Bulletin of the World Health Organization, 2008. **86**(5): p. 339-343.
26. Theodoratou, E., et al., *The effect of case management on childhood pneumonia mortality in developing countries.* International journal of epidemiology, 2010. **39**(suppl_1): p. i155-i171.
27. Lewin, S., et al., *Lay health workers in primary and community health care for maternal and child health and the management of infectious diseases.* Cochrane database of systematic reviews, 2010(3).
28. Young, M., et al., *World Health Organization/United Nations Children's Fund joint statement on integrated community case management: an equity-focused strategy to improve access to essential treatment services for children.* The American journal of tropical medicine and hygiene, 2012. **87**(5 Suppl): p. 6.
29. Sousa, A.d., et al., *Community case management of childhood illnesses: policy and implementation in Countdown to 2015 countries.* Bulletin of the World Health Organization, 2012. **90**(3): p. 183-190.
30. Mukanga, D., et al., *Can lay community health workers be trained to use diagnostics to distinguish and treat malaria and pneumonia in children? Lessons from rural Uganda.* Tropical Medicine & International Health, 2011. **16**(10): p. 1234-1242.
31. Kalyango, J.N., et al., *Performance of community health workers under integrated community case management of childhood illnesses in eastern Uganda.* Malaria Journal, 2012. **11**: p. 1-13.
32. Hamer, D.H., et al., *Quality and safety of integrated community case management of malaria using rapid diagnostic tests and pneumonia by community health workers.* Pathogens and Global Health, 2012. **106**(1): p. 32-39.
33. Gilroy, K.E., et al., *Quality of sick child care delivered by Health Surveillance Assistants in Malawi.* Health policy and planning, 2013. **28**(6): p. 573-585.
34. Lin, C., P. Tu, and L.M. Beitsch, *Confidence and receptivity for COVID-19 vaccines: a rapid systematic review.* Vaccines, 2020. **9**(1): p. 16.

35. Moher, D., et al., *Preferred reporting items for systematic reviews and meta-analyses: the PRISMA statement.* PLoS medicine, 2009. **6**(7): p. e1000097.
36. Labena, F., *Prevalence of Cerebral Palsy, Its Associated Factors And Mri Patho-Anatomic Findings Among Patients Attending Pediatric Neurology Clinic In Tikur Anbessa Specialized Hospital By: .* 2021.
37. Kakooza-Mwesige, A., *Cerebral palsy in Mulago hospital, Uganda: comorbidity, diagnosis and cultural adaptation of an assessment tool*. 2016: Karolinska Institutet (Sweden).
38. Ngassa, E.A., *Assessment of prevalence and risk factors for cerebral palsy: A case of Mbeya zonal referral hospital*. 2018, The Open University of Tanzania.
39. Kakooza-Mwesige, A., et al., *Prevalence of cerebral palsy in Uganda: a population-based study.* The Lancet Global Health, 2017. **5**(12): p. e1275-e1282.
40. Karumuna, J. and C. Mgone, *Cerebral palsy in Dar Es Salaam.* Central african journal of medicine, 1990. **36**(1): p. 8-10.
41. El-Tallawy, H.N., et al., *Cerebral palsy in Al-Quseir City, Egypt: prevalence, subtypes, and risk factors.* Neuropsychiatric Disease and Treatment, 2014: p. 1267-1272.
42. Tsige, S., et al., *Cerebral palsy in children: subtypes, motor function and associated impairments in Addis Ababa, Ethiopia.* BMC pediatrics, 2021. **21**: p. 1-11.
43. Coombe, H.J., *The clinical presentation of Cerebral Palsy in children in rural Kwazulu-Natal, South Africa.* 2017.
44. Salih, K., *Pattern of cerebral palsy among Sudanese children less than 15 years of age.* Cureus, 2020. **12**(3).
45. Couper, J., *Prevalence of childhood disability in rural KwaZulu Natal.* South African Medical Journal, 2002. **92**(7): p. 549-552.
46. El-Tallawy, H.N., et al., *Epidemiology of cerebral palsy in El-Kharga District-New Valley (Egypt).* Brain and Development, 2011. **33**(5): p. 406-411.
47. Abas, O., F. Abdelaziem, and A. Kilany, *Clinical spectrum of cerebral palsy and associated disability in South Egypt: a local survey study.* Open access Macedonian journal of medical sciences, 2017. **5**(1): p. 37.
48. Mangamba, D.K., et al., *Epidemiological, clinical, and treatment-related features of children with cerebral palsy in Cameroon: A hospital-based study.* Archives de Pédiatrie, 2022. **29**(3): p. 219-224.
49. Duke, R., et al., *Clinical features and aetiology of cerebral palsy in children from Cross River State, Nigeria.* Archives of Disease in Childhood, 2020. **105**(7): p. 625-630.
50. United Nations Inter-agency Group for Child Mortality Estimation (UN IGME), *Levels & Trends in Child Mortality: Report 2017*, in *UNICEF*. 2017: New York.
51. World Health Organization. *Diarrheal Disease*. 2023; Available from: http://www.who.int/news-room/fact-sheets/detail/diarrhoeal-disease.
52. Othero, D.M., et al., *Home management of diarrhea among underfives in a rural community in Kenya: household perceptions and practices.* East Afr J Public Health, 2008. **5**(3): p. 142-6.
53. Ashraf, S., et al., *Knowledge, attitude, and practice of mothers regarding home-based management of acute diarrhea in children under 5 years in selected private and public hospitals of Peshawar.* Journal of Rehman Medical Institute, 2019. **5**(4): p. 13-19.

54. Momoh, F.E., et al., *Mothers' knowledge, attitude and home management of diarrhoea among children under five years old in Lagos, Nigeria.* African Journal of Primary Health Care & Family Medicine, 2022. **14**(1): p. 3119.
55. Abdulla, O.N., et al., *Mothers Knowledge, Attitude and Practice Regarding Diarrhea and its Management in Aden-Yemen: A Cross-Sectional Study in Poor Resource Setting.* Journal of Pharmaceutical Research International, 2021. **33**(45B): p. 365-378.
56. Mallick, A.K., et al., *Knowledge, attitude and practice of acute diarrheal disease and use of oral rehydration therapy among mothers of under five children.* 2019.
57. Bauleth, M., H. Mitonga, and L. Pinehas, *Mothers'/caregivers' knowledge on aetiology, prevention and management of acute diarrhoea among children under 5 years in Engela District, Namibia.* Southern African Journal of Public Health, 2022: p. 19-28.
58. Sa'ad, Z.S.i., K.E. Hoque, and B.M. Arkilla, *Mothers' knowledge practices in home management of childhood diarrhea in kano state: A cross sectional study.* 2018.
59. Khaliq, A., et al., *Knowledge and practices on the prevention and management of diarrhea in children under-2 years among women dwelling in urban slums of Karachi, Pakistan.* Maternal and child health journal, 2022. **26**(7): p. 1442-1452.
60. He, Z., et al., *Factors influencing health knowledge and behaviors among the elderly in rural China.* International journal of environmental research and public health, 2016. **13**(10): p. 975.

Click here to access/download
Supporting Information
Supplementary Files.docx

PRISMA

Click here to access/download

Supporting Information

PRISMA_2020_checklist.pdf
