# Supplementary material for: Knowledge, attitude and practice of home management of diarrhea among under-five children in East Africa: A systematic review and meta-analysis
Source: PLoS One. 2024 Feb 23;19(2):e0298801. doi: 10.1371/journal.pone.0298801 (PMC10890776; doi:10.1371/journal.pone.0298801)
Supplement: Attachment — Submitted filename: Major comments.docx [file pone.0298801.s009.docx]

Dear authors, I appreciate you for giving me a chance to read your manuscript addressing an important and concerning topic. The manuscript was a good attempt at a crucial topic. I would like to suggest some changes that would enhance the quality of your work, and contribute to the scientific knowledge you want to write.

Major changes:

1. The manuscript mentions East Africa, however throughout the manuscript, authors have used the word Africa as a whole. This fails to clarify their intent behind writing. In the final analysis, authors include countries which are outside the boundaries of ‘East’ Africa,
2. Authors have made a blinding mistake of inserting a study characteristics table which is on ‘Cerebral Palsy’ instead of the relevant topic at hand.
3. Authors have been reckless with their grammar, 80% of the time was spent correcting the grammar. There was no proof reading done before submission, and it shows.
4. Figure 1 is also missing, the figures start from figure number 2.
5. It was painful to see the amount of repetition this manuscript had. Authors were going in circles trying to mention just the mortality caused by the diarrhea. They failed to mention anything else. This manuscript does not talk about anything else. There was an interesting link between co-packaging zinc with ORS, however authors forgot about that and abandoned that just as abruptly as they introduced it in the article.
6. Lack of funnel plot and subgroup analysis on South Sudan and Uganda.
7. Discussion lacked the knowledge gap. Failed to discuss the significance of results, and did not link all the manuscript together.
8. No mention of limitations of study.
9. Conclusion needs to be more descriptive as to what should be done.

Minor changes:

I have mentioned the rest of the comments within the manuscript, kindly look at them. The changes that were made by the reviewer were highlighted in yellow. Changes that need to be made are within notes marked as comments.

In my opinion, the authors can improve and work on this interesting topic, however, they will need to work on it heavily, especially the purpose of what they write. They need to go over redundancy and stop quoting the same bit of information a thousand times.
